# Biomass data for young, planted Norway spruce (*Picea abies* (L.) Karst.) trees in Eastern Carpathians of Romania

**DOI:** 10.1016/j.dib.2018.07.033

**Published:** 2018-07-19

**Authors:** Ioan Dutcă

**Affiliations:** a Transilvania University of Brasov, Romania; b Buckinghamshire New University, United Kingdom

## Abstract

Tree biomass data are essential for developing the biomass allometric models that are necessary for estimating carbon stock and for monitoring changes in forest biomass. In this ‘data article’ biomass records are presented for 240 Norway spruce trees (*Picea abies* (L.) Karst.). Trees were between 4 and 15 years of age and were sampled from 24 pure plantations located in Eastern Carpathians of Romania. Ten trees were sampled from each plantation using a cluster sampling method. For each tree, diameter at root collar height (D) and tree height (H) are provided as potential predictors for biomass. Oven-dried biomass is also recorded for the following partitions: stem (ST); branches (BR); needles (ND); roots (RT); as well as their combinations representing total aboveground biomass (AGB) and overall tree biomass (TB). Sampled trees were between 0.6 and 10.0 cm in diameter and between 53.0 and 552.0 cm in height. Total tree biomass ranged between 0.019 and 15.53 kg/tree. This dataset is related to the research article entitled “Site-effects on biomass allometric models for early growth plantations of Norway spruce (Picea abies (L.) Karst.)” (Dutcă et al., 2018) [1].

**Specifications Table**

**Table:** 

Subject area	*Forestry*
More specific subject area	*Estimation of tree biomass*
Type of data	*Table*
How data was acquired	*Data was acquired through measurement. The biomass of entire tree (including roots) was oven dried and then weighed. Precision of measurement is 1 mm for diameter, 1 cm for height and 0.1 g for biomass.*
Data format	*Raw*
Experimental features	*The data were collected from 24 plantations, and 10 trees sampled from each plantation.*
Data source location	*Location: Eastern Carpathians; Country: Romania*
	*Latitude: 45.44°N to 47.76°N*
	*Longitude: 24.96°E to 26.36°E*
Data accessibility	*Data is available with this article.*
Related research article	*Site-effects on biomass allometric models for early growth plantations of Norway spruce (Picea abies (L.) Karst.)*[1]

**Value of the data**

• The data can be used to develop biomass allometric models, biomass expansion factors and root to shoot ratios, in order to predict tree biomass.
• The data can be used to assess site-effects on biomass allometric models, and variance partitioning within and between forest stands.
• The data can be used to validate other biomass allometric models.
• The data on partitioning of biomass (stem, branch, needle, root) can be used to assess allocation to different tree components and in calibration of growth models.
• Combined with other datasets from a different region, the data can be used to assess the effect of the region on allometric models.
• The data can be used with other datasets to develop generic biomass allometric models.

## 1 Data

This ‘data article’ presents biomass records for 240 Norway spruce (*Picea abies* (L.) Karst.) trees sampled from 24 plantations. Ten trees were sampled from each plantation. The sampled trees were young (up to 15 years of age), therefore, their size was generally small. The diameter at root collar height (D) ranged from 0.6 to 10.0 cm and the tree height (H) from 53.0 to 552.0 cm. The total biomass of sampled trees ranged from 0.019 to 15.53 kg/tree (Table 1 and Supplementary material).

**Table 1:** Biomass of stem (ST), branches (BR), needles (ND), roots (RT), aboveground biomass (AGB, calculated as the sum of ST, BR, ND) and total tree biomass (TB, calculated as the sum of AGB and RT), for each tree in each forest stand, expressed in grams (g). Diameter at collar height (D) and tree height (H) are expressed in cm. The ‘Plantation’ defines the association of sampled trees in plantations.

Tree no.	Plantation	D (cm)	H (cm)	TB (g)	AGB (g)	ST (g)	BR (g)	ND (g)	RT (g)
1	1	0.9	69.0	37.5	29.8	12.2	6.7	10.9	7.7
2	1	0.9	68.0	46.0	37.4	13.3	8.1	16.0	8.6
3	1	0.9	65.0	39.2	33.0	10.3	8.0	14.7	6.2
4	1	0.9	64.0	35.3	26.2	9.2	6.2	10.8	9.1
5	1	1.0	67.0	55.8	45.0	15.8	10.9	18.3	10.8
6	1	1.0	66.0	43.7	37.3	13.2	7.4	16.7	6.4
7	1	1.0	68.0	40.2	33.7	12.4	7.1	14.2	6.5
8	1	1.0	70.0	42.8	36.3	14.8	8.1	13.4	6.5
9	1	1.0	67.0	46.5	36.8	14.4	9.7	12.7	9.7
10	1	1.1	67.0	57.0	47.6	13.9	12.4	21.3	9.4
11	2	0.6	56.0	19.5	14.5	5.1	2.4	7.0	5.0
12	2	0.6	56.0	24.9	17.1	5.4	2.5	9.2	7.8
13	2	0.6	55.0	24.1	18.4	6.0	3.5	8.9	5.7
14	2	0.6	53.0	24.5	16.0	4.8	2.9	8.3	8.5
15	2	0.7	54.0	20.4	15.8	6.0	2.4	7.4	4.6
16	2	0.7	57.0	39.7	28.3	7.9	6.3	14.1	11.4
17	2	0.7	57.0	24.1	18.0	5.8	3.9	8.3	6.1
18	2	0.7	58.0	28.0	22.3	6.4	4.2	11.7	5.7
19	2	0.8	55.0	32.8	22.5	6.5	5.0	11.0	10.3
20	2	0.8	58.0	46.5	34.4	7.8	6.9	19.7	12.1
21	3	1.0	78.0	75.4	61.0	20.2	11.8	29.0	14.4
22	3	1.0	76.0	47.0	37.5	16.9	6.2	14.4	9.5
23	3	1.1	78.0	70.0	53.9	23.0	12.2	18.7	16.1
24	3	1.1	78.0	65.6	50.3	22.3	9.9	18.1	15.3
25	3	1.1	77.0	82.9	67.2	24.9	15.0	27.3	15.7
26	3	1.1	74.0	55.7	41.3	19.2	7.5	14.6	14.4
27	3	1.1	80.0	66.9	54.4	22.7	11.5	20.2	12.5
28	3	1.2	83.0	72.5	57.3	29.5	13.7	14.1	15.2
29	3	1.2	78.0	84.7	69.1	22.2	17.0	29.9	15.6
30	3	1.2	81.0	71.4	58.1	24.3	13.4	20.4	13.3
31	4	1.1	72.0	74.5	64.3	22.5	15.2	26.6	10.2
32	4	1.2	73.0	88.5	73.8	23.9	19.2	30.7	14.7
33	4	1.2	70.0	90.6	70.1	26.1	17.8	26.2	20.5
34	4	1.3	71.0	105.3	86.3	25.8	22.0	38.5	19.0
35	4	1.3	72.0	117.3	92.4	24.4	25.8	42.2	24.9
36	4	1.3	75.0	87.0	71.2	26.1	15.9	29.2	15.8
37	4	1.4	73.0	92.5	75.5	26.7	17.2	31.6	17.0
38	4	1.4	72.0	118.3	97.1	30.3	26.6	40.2	21.2
39	4	1.4	74.0	94.4	78.3	29.2	18.9	30.2	16.1
40	4	1.5	72.0	101.7	84.8	26.4	21.7	36.7	16.9
41	5	1.2	87.0	117.4	99.0	28.8	25.1	45.1	18.4
42	5	1.3	92.0	104.4	85.8	32.0	19.5	34.3	18.6
43	5	1.3	84.0	115.1	95.0	29.9	22.4	42.7	20.1
44	5	1.3	86.0	140.1	110.1	35.7	21.4	53.0	30.0
45	5	1.3	81.0	113.4	91.1	31.6	19.7	39.8	22.3
46	5	1.4	87.0	109.5	82.7	32.3	18.5	31.9	26.8
47	5	1.4	86.0	111.0	84.1	36.2	17.1	30.8	26.9
48	5	1.5	88.0	122.2	106.1	31.0	25.3	49.8	16.1
49	5	1.5	87.0	155.7	118.2	39.4	30.3	48.5	37.5
50	5	1.5	88.0	143.2	109.2	43.4	23.0	42.8	34.0
51	6	1.6	108.0	195.0	148.6	51.9	42.4	54.3	46.4
52	6	1.7	104.0	242.7	184.1	56.2	55.6	72.3	58.6
53	6	1.8	115.0	225.8	183.2	57.1	47.0	79.1	42.6
54	6	1.8	113.0	216.0	177.3	58.6	41.9	76.8	38.7
55	6	1.8	102.0	292.9	224.7	70.4	60.2	94.1	68.2
56	6	1.8	112.0	173.8	141.3	55.9	37.9	47.5	32.5
57	6	1.8	109.0	231.4	183.7	65.5	48.4	69.8	47.7
58	6	1.9	111.0	222.6	176.9	61.3	54.4	61.2	45.7
59	6	1.9	102.0	213.8	162.6	59.7	47.5	55.4	51.2
60	6	2.0	104.0	268.8	206.6	65.5	60.3	80.8	62.2
61	7	1.5	88.0	140.2	122.0	33.7	33.9	54.4	18.2
62	7	1.6	87.0	166.2	140.9	39.6	41.9	59.4	25.3
63	7	1.7	94.0	202.8	173.3	44.4	48.7	80.2	29.5
64	7	1.7	95.0	168.6	140.5	47.2	38.1	55.2	28.1
65	7	1.8	95.0	179.0	150.5	52.6	48.7	49.2	28.5
66	7	1.8	92.0	173.1	145.9	42.8	39.0	64.1	27.2
67	7	1.9	95.0	249.4	210.4	54.0	56.5	99.9	39.0
68	7	1.9	97.0	269.8	223.7	52.5	66.8	104.4	46.1
69	7	1.9	92.0	193.3	159.6	52.5	45.9	61.2	33.7
70	7	2.0	93.0	229.3	194.6	53.7	52.2	88.7	34.7
71	8	1.4	85.0	107.5	92.6	31.2	17.1	44.3	14.9
72	8	1.4	83.0	150.8	107.9	41.5	24.5	41.9	42.9
73	8	1.4	80.0	138.1	116.3	34.0	26.0	56.3	21.8
74	8	1.4	77.0	91.2	71.5	24.1	15.0	32.4	19.7
75	8	1.4	77.0	107.2	84.5	30.3	15.2	39.0	22.7
76	8	1.6	85.0	104.9	84.4	33.1	17.3	34.0	20.5
77	8	1.6	82.0	143.7	119.5	43.7	26.2	49.6	24.2
78	8	1.6	85.0	122.7	97.7	37.9	19.9	39.9	25.0
79	8	1.6	81.0	132.9	102.0	35.9	19.6	46.5	30.9
80	8	1.8	81.0	99.6	78.1	32.7	13.6	31.8	21.5
81	9	1.4	68.0	126.9	100.9	30.4	23.5	47.0	26.0
82	9	1.5	75.0	142.4	123.0	34.0	29.4	59.6	19.4
83	9	1.5	71.0	157.4	126.1	43.3	27.7	55.1	31.3
84	9	1.6	68.0	187.9	148.8	46.2	41.0	61.6	39.1
85	9	1.6	71.0	172.7	138.0	37.6	34.9	65.5	34.7
86	9	1.7	70.0	197.3	158.0	49.7	37.1	71.2	39.3
87	9	1.7	72.0	160.0	130.4	40.1	29.8	60.5	29.6
88	9	1.7	70.0	198.7	160.0	44.5	40.8	74.7	38.7
89	9	1.8	72.0	206.8	162.1	41.2	45.1	75.8	44.7
90	9	1.9	76.0	184.6	154.9	52.9	36.3	65.7	29.7
91	10	2.0	120.0	291.0	245.5	79.3	63.8	102.4	45.5
92	10	2.0	125.0	342.3	290.4	79.4	81.4	129.6	51.9
93	10	2.0	119.0	292.6	234.2	84.8	48.6	100.8	58.4
94	10	2.1	121.0	379.1	306.9	82.3	77.3	147.3	72.2
95	10	2.1	121.0	285.4	231.9	73.8	49.0	109.1	53.5
96	10	2.2	121.0	427.5	345.1	102.4	88.1	154.6	82.4
97	10	2.3	125.0	397.6	325.2	100.9	87.4	136.9	72.4
98	10	2.3	126.0	397.1	308.7	99.0	71.1	138.6	88.4
99	10	2.3	122.0	400.1	320.2	94.4	82.7	143.1	79.9
100	10	2.4	127.0	415.5	338.1	108.3	77.5	152.3	77.4
101	11	2.2	97.0	416.9	323.7	74.9	83.9	164.9	93.2
102	11	2.2	105.0	366.6	304.6	73.3	74.9	156.4	62.0
103	11	2.2	107.0	495.5	378.6	87.6	100.2	190.8	116.9
104	11	2.3	101.0	468.7	353.2	88.6	93.2	171.4	115.5
105	11	2.3	103.0	426.5	329.7	91.7	88.8	149.2	96.8
106	11	2.4	104.0	539.1	440.5	84.0	120.1	236.4	98.6
107	11	2.4	104.0	395.5	310.4	81.0	72.2	157.2	85.1
108	11	2.5	98.0	545.9	435.7	96.7	125.0	214.0	110.2
109	11	2.5	101.0	433.2	333.5	81.8	88.0	163.7	99.7
110	11	2.7	102.0	525.5	440.0	93.8	121.8	224.4	85.5
111	12	2.3	110.0	426.7	362.9	96.9	77.5	188.5	63.8
112	12	2.4	114.0	530.8	444.2	114.0	92.6	237.6	86.6
113	12	2.4	111.0	491.2	414.0	115.4	95.0	203.6	77.2
114	12	2.5	108.0	395.0	255.1	104.6	58.9	139.9	91.6
115	12	2.6	108.0	502.2	408.3	98.4	102.1	207.8	93.9
116	12	2.6	115.0	401.3	333.7	111.7	68.4	153.6	67.6
117	12	2.6	110.0	422.8	332.9	117.3	64.9	150.7	89.9
118	12	2.6	111.0	471.1	377.7	122.6	94.1	161.0	93.4
119	12	2.6	108.0	501.4	408.8	104.6	108.8	195.4	92.6
120	12	2.6	107.0	505.9	425.7	103.6	100.5	221.6	80.2
121	13	2.7	121.0	653.5	513.3	127.6	134.4	251.3	140.2
122	13	2.7	120.0	667.9	561.7	129.8	155.3	276.6	106.2
123	13	2.7	122.0	716.5	579.7	140.7	165.8	273.2	136.8
124	13	2.7	119.0	620.6	479.6	116.9	136.8	225.9	141.0
125	13	2.8	119.0	811.3	678.2	128.4	221.5	328.3	133.1
126	13	2.8	121.0	560.8	435.3	107.5	118.3	209.5	125.5
127	13	2.8	123.0	815.8	649.2	129.8	187.3	332.1	166.6
128	13	2.9	124.0	606.0	500.1	117.9	130.7	251.5	105.9
129	13	3.1	121.0	783.3	645.0	112.6	198.2	334.2	138.3
130	13	3.1	121.0	628.8	539.7	126.5	136.5	276.7	89.1
131	14	3.1	166.0	899.1	722.7	195.4	215.8	311.5	176.4
132	14	3.2	155.0	870.3	727.3	189.1	192.3	345.9	143.0
133	14	3.3	165.0	1026.0	783.8	209.5	216.6	357.7	242.2
134	14	3.3	165.0	791.5	646.7	226.7	152.6	267.4	144.8
135	14	3.4	160.0	908.7	734.2	182.2	204.6	347.4	174.5
136	14	3.5	164.0	1076.0	805.1	229.8	228.8	346.5	270.9
137	14	3.5	171.0	981.9	734.2	222.6	183.9	327.7	247.7
138	14	3.5	164.0	972.5	744.2	214.8	221.9	307.5	228.3
139	14	3.5	160.0	1081.8	876.9	198.9	245.9	432.1	204.9
140	14	3.9	175.0	1128.1	935.8	252.8	306.6	376.4	192.3
141	15	3.8	226.0	1825.5	1539.2	358.0	452.4	728.8	286.3
142	15	3.9	231.0	1869.0	1566.9	410.1	560.9	595.9	302.1
143	15	4.1	231.0	1727.9	1466.7	456.6	408.0	602.1	261.2
144	15	4.1	232.0	1808.5	1467.0	373.2	440.9	652.9	341.5
145	15	4.1	236.0	1920.0	1625.3	525.6	460.8	638.9	294.7
146	15	4.2	238.0	1878.9	1575.2	435.9	524.6	614.7	303.7
147	15	4.2	231.0	1886.3	1569.8	420.8	438.8	710.2	316.5
148	15	4.3	234.0	1983.0	1691.9	438.6	444.7	808.6	291.1
149	15	4.3	229.0	1878.2	1500.2	449.6	441.5	609.1	378.0
150	15	4.5	236.0	2449.0	2142.8	532.3	657.4	953.1	306.2
151	16	2.7	148.0	638.3	445.1	125.3	125.8	194.0	193.2
152	16	2.7	148.0	843.6	663.2	134.9	231.2	297.1	180.4
153	16	2.9	151.0	841.9	669.3	159.7	223.5	286.1	172.6
154	16	3.0	157.0	1007.5	800.1	189.8	271.9	338.4	207.4
155	16	3.0	151.0	699.4	530.6	138.7	156.8	235.1	168.8
156	16	3.0	155.0	752.0	580.5	137.2	182.6	260.7	171.5
157	16	3.1	153.0	975.8	759.0	176.7	283.0	299.3	216.8
158	16	3.2	168.0	917.7	759.8	208.6	245.5	305.7	157.9
159	16	3.2	150.0	997.2	803.3	168.3	263.2	371.8	193.9
160	16	3.5	154.0	758.4	574.9	199.4	160.0	215.5	183.5
161	17	5.0	305.0	3139.2	2707.5	811.6	737.6	1158.3	431.7
162	17	5.2	301.0	3746.2	3147.6	945.4	896.2	1306.0	598.6
163	17	5.3	304.0	3506.4	2875.5	838.5	853.7	1183.3	630.9
164	17	5.5	295.0	4007.0	3214.0	1099.4	836.8	1277.8	793.0
165	17	5.5	313.0	4127.6	3315.8	947.5	889.9	1478.4	811.8
166	17	5.7	306.0	3794.0	3179.6	1093.3	743.7	1342.6	614.4
167	17	5.7	302.0	4078.5	3266.9	919.4	889.6	1457.9	811.6
168	17	6.2	306.0	5022.6	4015.5	1210.3	1186.0	1619.2	1007.1
169	17	6.2	312.0	4876.5	3808.7	1056.5	1109.9	1642.3	1067.8
170	17	6.3	306.0	5218.0	4399.4	1240.4	1539.0	1620.0	818.6
171	18	7.4	361.0	5978.9	5589.5	1756.7	1481.7	2351.1	389.4
172	18	8.3	349.0	7330.6	6051.9	1632.2	1929.4	2490.3	1278.7
173	18	8.5	350.0	6279.0	5210.6	1902.4	1581.5	1726.7	1068.4
174	18	8.5	349.0	5625.1	4529.4	1833.7	1158.4	1537.3	1095.7
175	18	8.5	355.0	7689.3	6465.1	1913.0	2054.9	2497.2	1224.2
176	18	8.5	364.0	6462.5	5687.7	2023.0	1534.4	2130.3	774.8
177	18	8.5	350.0	5564.1	4865.8	1616.2	1317.0	1932.6	698.3
178	18	8.6	358.0	7356.2	6176.9	1894.2	2029.8	2252.9	1179.3
179	18	8.9	350.0	9220.3	8098.0	2340.4	2761.2	2996.4	1122.3
180	18	8.9	354.0	6307.3	5354.4	1947.0	1486.9	1920.5	952.9
181	19	4.5	220.0	2337.2	1828.8	387.0	683.0	758.8	508.4
182	19	4.6	224.0	2325.5	1869.2	474.3	557.1	837.8	456.3
183	19	4.7	218.0	1796.7	1524.3	398.7	573.7	551.9	272.4
184	19	4.8	210.0	1769.0	1374.1	382.5	469.5	522.1	394.9
185	19	4.9	215.0	2152.3	1768.3	477.9	660.3	630.1	384.0
186	19	5.0	216.0	2130.4	1642.6	502.1	556.4	584.1	487.8
187	19	5.2	221.0	2913.0	2326.5	645.4	822.0	859.1	586.5
188	19	5.4	216.0	2725.3	2270.5	578.1	853.1	839.3	454.8
189	19	5.8	221.0	2337.7	1943.5	620.9	614.1	708.5	394.2
190	19	5.8	220.0	2585.9	2093.5	538.2	733.7	821.6	492.4
191	20	5.8	317.0	3617.2	2991.2	886.6	968.1	1136.5	626.0
192	20	6.2	327.0	3966.5	3207.8	900.5	1002.9	1304.4	758.7
193	20	6.4	325.0	4063.1	3401.8	1023.8	1100.7	1277.3	661.3
194	20	6.4	320.0	4288.5	3482.5	1038.4	1096.5	1347.6	806.0
195	20	6.5	324.0	3932.4	3287.5	1089.7	1000.4	1197.4	644.9
196	20	6.6	331.0	4127.1	3480.6	948.9	1139.7	1392.0	646.5
197	20	6.6	326.0	4185.7	3497.6	1032.3	1037.5	1427.8	688.1
198	20	6.6	327.0	4179.4	3558.2	1121.1	1027.4	1409.7	621.2
199	20	6.7	321.0	3994.8	3235.7	1014.2	1024.1	1197.4	759.1
200	20	7.5	320.0	5775.8	4638.0	1269.8	1616.0	1752.2	1137.8
201	21	6.6	383.0	6408.2	5005.6	1312.6	1697.4	1995.6	1402.6
202	21	6.7	362.0	6055.6	4943.8	1239.2	1329.4	2375.2	1111.8
203	21	6.9	375.0	6117.8	4866.4	1433.6	1335.3	2097.5	1251.4
204	21	7.0	371.0	6084.6	4863.4	1320.3	1581.5	1961.6	1221.2
205	21	7.1	359.0	6235.0	4798.5	1281.9	1398.9	2117.7	1436.5
206	21	7.1	367.0	6344.9	5355.8	1521.7	1751.2	2082.9	989.1
207	21	7.2	375.0	6760.7	5703.9	1476.6	1762.1	2465.2	1056.8
208	21	7.5	370.0	6153.6	5220.6	1471.1	1820.8	1928.7	933.0
209	21	7.6	372.0	6744.2	5770.2	1586.5	1690.1	2493.6	974.0
210	21	8.5	350.0	6145.5	5049.3	1413.3	1431.9	2204.1	1096.2
211	22	6.0	347.0	3789.2	3329.2	1035.8	1106.6	1186.8	460.0
212	22	6.3	335.0	4067.0	3263.5	1034.8	1033.0	1195.7	803.5
213	22	6.4	348.0	4025.7	3283.9	1053.2	1081.7	1149.0	741.8
214	22	6.5	330.0	4179.7	3633.9	1113.1	1140.2	1380.6	545.8
215	22	6.5	334.0	4976.3	4144.4	1091.2	1378.5	1674.7	831.9
216	22	6.5	346.0	4550.4	3808.9	1078.3	1366.8	1363.8	741.5
217	22	6.8	357.0	4493.4	3835.4	1094.7	1304.4	1436.3	658.0
218	22	7.0	347.0	6887.8	5790.0	1475.2	1743.2	2571.6	1097.8
219	22	7.3	352.0	5589.1	4641.5	1453.8	1676.5	1511.2	947.6
220	22	7.4	349.0	4659.1	4025.6	1268.6	1312.7	1444.3	633.5
221	23	9.1	535.0	12359.8	10430.6	3371.6	3312.8	3746.2	1929.2
222	23	9.3	526.0	11721.7	9927.5	3999.3	2746.4	3181.8	1794.2
223	23	9.4	542.0	10537.6	9091.5	2883.6	2869.2	3338.7	1446.1
224	23	9.6	554.0	12955.3	11136.4	5881.6	2329.4	2925.4	1818.9
225	23	9.8	526.0	13839.0	11667.9	4268.2	3742.0	3657.7	2171.1
226	23	9.8	533.0	12327.4	10614.0	4148.2	2697.8	3768.0	1713.4
227	23	9.8	552.0	13883.4	12266.1	4790.1	3458.3	4017.7	1617.3
228	23	9.8	549.0	15382.8	13463.2	4276.9	4300.2	4886.1	1919.6
229	23	9.9	540.0	13674.7	11926.4	4892.4	3540.6	3493.4	1748.3
230	23	10.0	547.0	15531.2	13905.2	4149.2	3731.4	6024.6	1626.0
231	24	6.9	436.0	6140.7	5030.3	1677.3	1552.7	1800.3	1110.4
232	24	7.3	433.0	5689.7	4833.5	1847.2	1487.8	1498.5	856.2
233	24	7.4	420.0	5880.0	4918.4	1554.7	1475.3	1888.4	961.6
234	24	7.5	437.0	7657.5	5979.8	2269.2	1839.4	1871.2	1677.7
235	24	7.6	421.0	6208.0	4908.0	1958.2	1396.0	1553.8	1300.0
236	24	7.7	444.0	6862.3	5673.4	2126.2	1791.6	1755.6	1188.9
237	24	7.7	442.0	6743.9	5566.8	1970.0	1834.7	1762.1	1177.1
238	24	7.8	435.0	5923.9	4990.0	1765.3	1567.9	1656.8	933.9
239	24	7.8	439.0	5898.6	4850.9	1754.2	1375.5	1721.2	1047.7
240	24	8.5	440.0	7607.4	6413.7	2320.7	1994.0	2099.0	1193.7

The clustered structure of this dataset permitted the investigation of site-effects in allometric models, by separating the model variance produced by the variability of trees within plantations from the variance produced by variability between plantations [1]. Furthermore, part of this dataset was used to develop biomass conversion and expansion factors [2] and to assess the influence of age, latitude, altitude and soil properties on biomass allometric models [3].

## 2 Experimental design, materials, and methods

The trees were sampled from 24 plantations of pure Norway spruce in Eastern Carpathians of Romania, between 45.44°N and 47.76°N in latitude and between 24.96°E and 26.36°E in longitude (Table 2). The age of trees at the moment of sampling varied between 4 and 15 years (in each plantation, the trees had similar age). Age of trees (see Table 1, in Ref. [1]) was calculated as the difference between year of sampling and year of planting (provided by forest management plan), plus the number of years spent in forest nurseries (we adopted a standard number of 3 years for all sampled trees, as this is the usual practice for the species). The calculated age was then validated by counting the number of tree rings on sampled trees. Mean annual temperature (MAT) and mean annual precipitation (MAP) were derived from WorldClim, global climate data [4]. MAT varied between 2.6 and 7.3 °C and MAP ranged between 643.0 and 932.6 mm. Soil pH, base saturation and texture were based on sample soil taken from each plantation.

**Table 2:** Characteristics of sampled plantations.

Plantation no.	Latitude (North)	Longitude (East)	Mean annual temperature (°C)	Mean annual precipitation (mm)	Soil pH	Soil base saturation (%)	Soil texture^a^
1	47°35′15′′	25°00′41′′	4.4	827.3	6.24	72	L
2	45°31′98′′	25°57′55′′	3.9	869.7	4.36	37	SL
3	47°38′29′′	25°03′11′′	4.7	805.2	7.28	85	L
4	47°34′41′′	25°00′03′′	4.9	799.6	4.90	29	SL
5	47°29′39′′	25°35′58′′	5.9	705.4	5.94	70	SL
6	47°45′43′′	25°39′02′′	6.3	702.8	7.74	90	SL
7	45°31′28′′	25°53′64′′	4.0	872.8	5.33	59	LS
8	45°31′06′′	25°35′15′′	5.8	822.8	5.31	62	L
9	45°35′45′′	25°34′00′′	5.1	853.4	6.21	66	LS
10	45°26′25′′	25°32′54"	5.9	824.6	5.80	69	LS
11	45°26′48′′	25°34′01′′	5.9	814.9	6.15	43	SL
12	45°33′46′′	25°54′82′′	5.7	785.0	5.13	51	LS
13	45°26′48′′	25°34′03′′	5.9	815.9	6.15	43	SL
14	45°36′30′′	25°29′19′′	7.3	687.7	6.77	86	SL
15	45°27′07′′	25°34′15′′	5.4	855.1	5.18	52	L
16	47°02′57′′	25°49′12′′	6.0	664.8	5.62	76	L
17	46°57′37′′	25°29′24′′	4.8	750.5	5.73	49	SL
18	45°27′29′′	25°34′02′′	5.6	842.5	5.92	82	SL
19	47°35′40′′	24°57′35′′	2.6	932.6	5.48	58	L
20	47°36′08′′	24°59′12′′	4.5	821.0	4.92	47	SL
21	47°11′17′′	26°03′18′′	6.4	643.0	7.21	88	L
22	46°04′37′′	26°21′38′′	5.9	677.2	4.88	51	SL
23	45°26′36′′	25°32′56′′	5.8	827.6	5.80	69	L
24	47°35′40′′	24°57′35′′	4.8	808.6	5.68	70	LS

a L: Loam; SL: Sandy loam; LS: Loamy sand.

### 2.1 Sampling design

The tree selection in each plantation was meant to capture the entire variability of biomass-predictor, in order to assess the site-effects on biomass allometric models [1]. Biomass allometric models, which are regression models that use tree diameter and/or height to predict biomass, are influenced by tree architecture and wood density variation. These two characteristics (i.e. tree architecture and wood density) are modulated by genotype [5], tree competition [6] and environmental conditions [7]. Since wood density is inherently species specific and because the current dataset involves only one tree species, the sampling strategy has been focused on tree architecture only. Nevertheless, an important vector of tree architecture is the H–D ratio. Therefore, to capture the variability of H–D ratio in each plantation, H was set constant while D was let to vary. Diameter was let to vary, since D has greater effect on biomass compared to H [1]. Setting H constant was also helpful to facilitate the identification of candidate trees within plantation (as H is better visually approximated compared to D, especially in young stands).

### 2.2 Biomass measurements

**Fig. 1:**
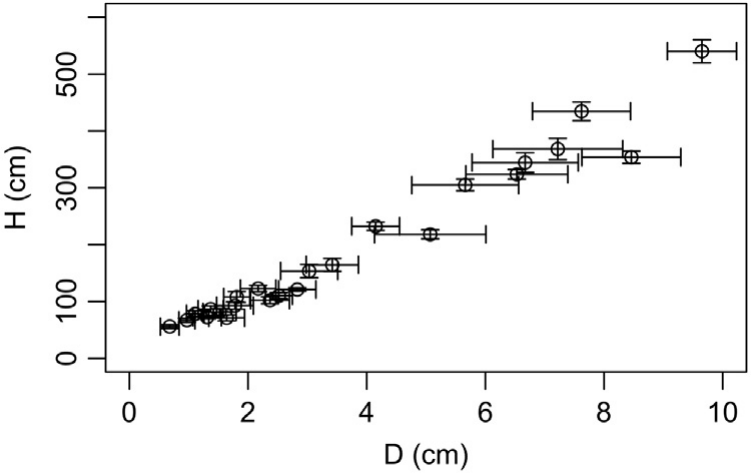
The variability of D and H for the sampled trees.

In each plantation a sample plot of 200 m^2^ was delimited and all trees were measured for D and H. The ‘mean height’ was calculated, as the height corresponding to the mean ‘collar area’ (similar to basal area but calculated based on root collar diameter). Ten trees in each plantation were selected and destructively sampled. The selection criteria were (i) H of candidate trees to be close to ‘mean height’ (calculated for each plantation) and (ii) D was let to vary (Fig. 1). Trees showing signs of affected structural integrity or disease were avoided.

A total of 240 trees were sampled, with D ranging from 0.6 to 10.0 cm, and H between 53.0 and 552.0 cm (Table 1). For each sampled tree, D and H were measured in situ. Fresh biomass of each tree was separated in 3 fractions: (i) stem, (ii) branches with attached needles and (iii) roots. As trees were generally small, biomass of entire tree was oven-dried in the laboratory. The branches with attached needles were pre-dried for 24 hours to ease separation of needles from wooden branch fraction. After separation, all tree biomass components (i.e. stem, branches, needles and roots) were dried until constant weight (for approximately 7 days) at 80 °C. All biomass fractions were weighed with an electronic scale (with a precision of ±0.1 g). Tree biomass components, as used in this article, are defined as: (i) stem – the aboveground axis of the tree, from the ground (including the stump) to the highest bud and excluding therefore the branches and needles; (ii) branches – the aboveground woody structures that were not included in ‘stem’ category; (iii) needles – the non-woody aboveground tree component, including the buds; (iv) roots (coarse roots) – the belowground biomass, defined by root diameter of at least 2.0 mm.

## References

[1] Dutcă I., Mather R., Blujdea V.N.B., Ioraș F., Olari M., Abrudan I.V. (2018). Site-effects on biomass allometric models for early growth plantations of Norway spruce (Picea abies (L.) Karst.). Biomass Bioenergy.

[2] Dutcă I., Abrudan I.V., Stăncioiu P.T., Blujdea V. (2010). Biomass conversion and expansion factors for young Norway Spruce (Picea abies (L.) Karst.) trees planted on non-forest lands in Eastern Carpathians. Not. Bot. Horti Agrobot. Cluj-Napoca.

[3] Dutcă I., Negruţiu F., Ioraş F., Maher K., Blujdea V.N.B., Ciuvăţ L.A., Negrutiu F., Ioras F., Maher K., Blujdea V.N.B., Ciuvat L.A. (2014). The influence of age, location and soil conditions on the allometry of young Norway spruce ( Picea abies L. Karst.) trees. Not. Bot. Horti Agrobot. Cluj-Napoca.

[4] Hijmans R.J., Cameron S.E., Parra J.L., Jones P.G., Jarvis A. (2005). Very high resolution interpolated climate surfaces for global land areas. Int. J. Climatol..

[5] Kroon J., Andersson B., Mullin T.J. (2008). Genetic variation in the diameter–height relationship in Scots pine (Pinus sylvestris). Can. J. Res..

[6] Dutcă I., Mather R., Ioraş F. (2018). Tree biomass allometry during the early growth of Norway spruce (Picea abies) varies between pure stands and mixtures with European beech (Fagus sylvatica). Can. J. Res..

[7] Poorter H., Niklas K.J., Reich P.B., Oleksyn J., Poot P., Mommer L. (2012). Biomass allocation to leaves, stems and roots: meta-analyses of interspecific variation and environmental control. New Phytol..

