# Supplementary material for: Biomass data for young, planted Norway spruce (Picea abies (L.) Karst.) trees in Eastern Carpathians of Romania
Source: Data Brief. 2018 Jul 19;19:2384–92. doi: 10.1016/j.dib.2018.07.033 (PMC6141963; doi:10.1016/j.dib.2018.07.033)
Supplement: Supplementary file 1 — Supplementary material [file mmc1.pdf]

Conflict of Interest and Authorship Conformation Form

Please check the following as appropriate:

- o All authors have participated in (a) conception and design, or analysis and interpretation of the data; (b) drafting the article or revising it critically for important intellectual content; and (c) approval of the final version.
- o This manuscript has not been submitted to, nor is under review at, another journal or other publishing venue.
- o The authors have no affiliation with any organization with a direct or indirect financial interest in the subject matter discussed in the manuscript
- o The following authors have affiliations with organizations with direct or indirect financial interest in the subject matter discussed in the manuscript:

Author's name	Affiliation
Ioan Dutca	Transilvania University of Brasov and Buckinghamshire New University
